# Introduction into natural environments shifts the gut microbiome of captivity-raised filter-feeding bivalves

**DOI:** 10.1093/ismeco/ycae125

**Published:** 2024-10-23

**Authors:** Stephanie N Vaughn, Garrett W Hopper, Irene Sánchez González, Jamie R Bucholz, Ryan C Garrick, Jeffrey D Lozier, Paul D Johnson, Carla L Atkinson, Colin R Jackson

**Affiliations:** Department of Biology, University of Mississippi, University, MS 38677, United States; School of Renewable Natural Resources, Louisiana State University and Agricultural Center, Baton Rouge, LA 70803, United States; Odum School of Ecology, University of Georgia, Athens, GA 30602, United States; Department of Biological Sciences, University of Alabama, Tuscaloosa, AL 35487, United States; Department of Biology, University of Mississippi, University, MS 38677, United States; Department of Biological Sciences, University of Alabama, Tuscaloosa, AL 35487, United States; Alabama Department of Conservation and Natural Resources, Alabama Aquatic Biodiversity Center, Marion, AL 36756, United States; Department of Biological Sciences, University of Alabama, Tuscaloosa, AL 35487, United States; Department of Biology, University of Mississippi, University, MS 38677, United States

**Keywords:** species introductions, freshwater mussels, gut microbiome, environment, conservation

## Abstract

The gut microbiome is influenced by host species and the environment, but how the environment influences the microbiome of animals introduced into a new ecosystem has rarely been investigated. Freshwater mussels are aquatic fauna, with some threatened or endangered species propagated in hatcheries and introduced into natural systems as part of conservation efforts. The effects of the environment on the freshwater mussel gut microbiome were assessed for two hatchery-propagated species (*Lampsilis ovata*, *Lampsilis ornata*) introduced into rivers within their natural range. Mussels were placed in rivers for 8 weeks, after which one subset was collected, another subset remained in that river, and a third subset was reciprocally transplanted to another river in the same river basin for a further 8 weeks. Gut microbiome composition and diversity were characterized for all mussels. After the initial 8 weeks, mussels showed increased gut bacterial species richness and distinct community composition compared to hatchery mussels, but gut microbiome diversity then decreased for mussels that remained in the same river for all 16 weeks. The gut bacterial community of mussels transplanted between rivers shifted to resemble that of mussels placed initially into the recipient river and that remained there for the whole study. All mussels showed high proportions of *Firmicutes* in their gut microbiome after 8 weeks, suggesting an essential role of this phylum in the gut of *Lampsilis* species. These findings show that the mussel gut microbiome shifts in response to new environments and provide insights into conservation strategies that involve species reintroductions.

## Introduction

Freshwater unionid mussels are a diverse and imperiled group of filter-feeders that perform important ecosystem functions connecting benthic and pelagic food webs [1–6]. However, over 40% of freshwater mussels are threatened, endangered, or extinct, with more declines projected [7, 8]. Dramatic and “enigmatic” [9] declines in North American freshwater mussels occurred from 1970 to 1990 and revealed their sensitivity to environmental change and anthropogenetic pressures [5, 10–13]. Understanding how the environment influences the biology of these mussels is critical for devising effective conservation strategies, and their gut microbial community is likely to be particularly sensitive to environmental change [13].

Changing environmental conditions can lead to gut dysbiosis and increase host susceptibility to pathogens [14–18], and one hypothesis explaining the decline of North American freshwater mussels has been the emergence of an unknown pathogen [19, 20], an idea supported by recent studies that have reported pathogenic bacteria and viruses in moribund pheasantshell (*Actinonaias pectorosa*) mussels [18, 21]. However, freshwater mussels also maintain a natural gut bacterial community that can be species-specific [22, 23], vary spatially and temporally [22, 24, 25], and that is distinct from that on suspended seston [22, 26]. For one mussel species, the gut bacterial community differs between wild and captive individuals, with captive mussels having lower gut diversity that may reduce their overall health [17, 27]. As conservation efforts include reintroducing hatchery-propagated mussels into their natural environments [28–30], it is important to understand how such captivity-raised mussels respond to an environment with more diverse food sources and more fluctuating environmental conditions than where they developed. Placing such hatchery-propagated mussels into wild rivers also provides a rare opportunity to examine how the environment shapes the microbiome of freshwater bivalves. While changes in the gut microbiome following placing wild animals into captivity have been examined for invertebrates, fish, and birds [17, 31–33], few studies have examined the effects when placing organisms raised in captivity into natural environments.

In this study, we investigated the influence of the environment on the gut microbiome of two species of hatchery-propagated freshwater mussels introduced into natural rivers. Mussels were placed in “silos,” enclosures that caged mussels in rivers to allow later collection while allowing exchange between the mussels and the surrounding environment [5, 30, 34]. These silos also allowed the transplant of mussels between rivers to further determine the effects of environmental conditions on the gut microbiome of animals released from captivity into natural ecosystems. The bacterial gut microbiome of these mussels was characterized after 8 weeks in the initial recipient river and 8 weeks later, after a subset of silos had been transplanted to a different river. We predicted that the gut microbiome of these hatchery-propagated animals would shift when exposed to natural environmental conditions, with increases in gut bacterial diversity through recruitment from bacterial communities in surrounding water and sediment. Since environmental conditions and microbiomes could help shape the mussel gut microbiome, we predicted that transplants between rivers would lead to further shifts in gut bacterial composition, with the gut microbiomes of transplanted mussels approaching those of mussels placed in the receiving river initially.

## Materials and methods

### Hatchery-propagated mussels

*Lampsilis ovata* (native to the Tennessee River Basin; TRB) and *Lampsilis ornata* (native to the Mobile River Basin; MRB) were reared at the Alabama Aquatic Biodiversity Center (AABC; Marion, AL, USA) to a mean length of 25.1 mm and 31.2 mm, respectively. As well as mussels introduced into rivers (see below), eight mussels of each species were collected prior to the experiment to determine initial gut microbiome diversity and composition, and a further eight mussels collected at the end of the experiment (16 weeks) from those that remained in the hatchery. Samples of seston (1 L water filtered through 0.7-μm filters; *n* = 3) and sediment (1.0 g, *n* = 3) were collected from hatchery ponds prior to, halfway through (8 weeks), and at the end of the experiment to assess hatchery environmental bacterial communities.

### Experimental design

Mussels were placed into rivers in concrete silos (240 mm diameter, 150 mm high), containing an internal PVC chamber (100 mm diameter) with 6 mm mesh coverings on the top and bottom. Each silo had three external support feet to keep the silos 30 mm above the riverbed and allow for water flow through the internal chamber [30, 35].

Nine silos, each containing 5–6 mussels, were placed into each of the Duck and Paint Rock rivers of the TRB (*L. ovata*; May 2022) and the Sipsey and Cahaba rivers of the MRB (*L. ornata*; June 2022). After 57 days (8 weeks), three silos were left in place at each site, three silos were removed from the river and mussels collected, and three silos were transplanted to the reciprocal site of each river basin (from the Duck to the Paint Rock River, and vice versa, and from the Sipsey to the Cahaba River, and vice versa). After 112 days (16 weeks), all silos were removed from rivers and mussels collected.

HOBO data loggers (Onset, Cape Cod, MA) measured water temperature and conductivity hourly throughout the experiment. Every 2–3 weeks, sediment and seston were collected from each site and processed as were hatchery samples, and mussel shell lengths were measured using calipers. Water samples were collected every 2–3 weeks to determine soluble reactive phosphorous (SRP) colorimetrically [36, 37] and NH_4_^+^ using the phenol method [37, 38] with a Seal AQ300 discrete analyzer.

### DNA extraction, amplification, and sequencing

Mussels were immediately frozen in liquid nitrogen and later thawed, and the gut was dissected and removed [23, 25, 26]. Gut samples were ground using sterile pellet pestles and the extraction buffer from a PowerSoil Pro kit (Qiagen, Germantown, MD, USA), followed by bacterial DNA extractions using the manufacturer’s vortex adapter and recommended protocol [26]. Samples of filtered seston and sediment were extracted directly with a PowerSoil Pro kit.

Amplifications targeted 250 base pairs (bp) of the V4 region of the bacterial 16S rRNA gene using dual-index 8-nucleotide barcoding and with a single round of PCR per sample [39]. Amplification products were normalized using SequalPrep plates (Life Technologies, Grand Island, NY, USA) and pooled prior to sequencing. All mussel samples (223) were pooled into one library, and the assembled library spiked with 20% PhiX [40, 41] and sequenced on an Illumina MiSeq at the University of Mississippi Medical Center Molecular and Genomics Core Facility. Seston and sediment samples (201) were prepared for sequencing the same way but sequenced later on an Illumina NextSeq.

Fastq files were processed using the standard pipeline of DADA2 v.1.26.0 [42] in R v.4.2.2. MiSeq sequences were trimmed/filtered using parameters: truncLen = c(240,160), maxN = 0, maxEE = c(2,2), truncQ = 2. NextSeq sequences were trimmed/filtered using parameters: truncLen = c(240,180), maxN = 0, maxEE = c(2,2), truncQ = 2. Quality profile plots were inspected to verify the quality of trimmed reads. The two sequencing runs were merged into one object using *mergeSequenceTables* in DADA2. Sequences were further trimmed to remove any overhang (trimOverhang = TRUE), and sequences <250 and >257 bp or identified as potential chimeras, chloroplasts, mitochondria, *Archaea*, or *Eukarya* were removed. Sequences were classified against RDP v.18 [43]. Final amplicon sequence variant (ASV) data were transformed into relative abundance (% sequence reads) of bacterial taxa for downstream analyses.

### Statistical analyses

Mussels grew consistently within each river and did not significantly differ based on river of placement; therefore, growth data was not utilized in statistical analyses. Differences in environmental factors between rivers were assessed using one-way ANOVA. Mantel correlations were conducted to examine relationships between seston and sediment bacterial community composition and environmental factors using Bray–Curtis dissimilarity scores and a distance matrix of each environmental variable over time.

Singleton ASVs were removed from the sequence dataset using *prune_taxa* from “phyloseq” [44] to reduce potential noise from rare taxa. Alpha diversity metrics were calculated with *estimate_richness* from “phyloseq.” Linear mixed-effects models were fixed with “lme4” [45] to determine influences of time and river on bacterial species richness (S_obs_) in the gut community. The silo number was set as a random effect to account for potential correlation among mussels within the same silo. To evaluate models for significance, Type III ANOVA from “car” [46] was used by employing Wald Chi-square tests to assess the overall contribution of each predictor. Post-hoc Tukey contrasts of linear mixed-effect models were performed with the Holm method using the *glht* function from “multcomp” [47].

Differences in the relative abundances of major bacterial phyla in the mussel gut microbiome between river of placement and time in each river (“status”; 0-, 8-, or 16-week) were determined using MANOVA. Phyla were investigated using Tukey’s HSD post-hoc tests when >2 predictor variables were present in the model (i.e. post-hoc comparisons between AABC and pairs of rivers). PERMANOVAs using Bray–Curtis dissimilarity scores were performed using *adonis2* in “vegan” [48] to determine differences in overall bacterial composition between sample type, river, and mussel status. Significant differences were assessed using “pairwise.adonis” [49] and visualized using non-metric multidimensional scaling (NMDS). The “strata” term was used when running PERMANOVAs to account for potential correlation between mussels within the same silo. The estimated influence of seston and sediment bacterial communities (sources) on the gut microbiome of mussels was determined using fast expectation–maximization for microbial source tracking (FEAST; [50]). Relative abundances of seston and sediment ASVs were separated into initial (samples from weeks 0 to 8) and final (samples from weeks 8 to 16) groups for each FEAST analysis to determine influence over time. The “core” microbiome of 16-week transplanted and non-transplanted mussels was determined using “prevalence” in “microbiome” [51]. This defined the “core” microbiome of mussel samples by calculating the relative abundance of ASVs that comprised >1% of the total ASVs identified within each sample and ranking them based on relative frequency.

## Results

We used sequence data from 94 *L. ornata*, 94 *L. ovata*, 84 seston, and 82 sediment samples from rivers, and 16 *L. ornata*, 16 *L. ovata*, 18 seston, and 15 sediment samples from the hatchery (one *L. ovata*, two *L. ornata*, and two sediment samples were removed because they contained <600 valid sequences). The mean (±SE) sequence quality score was 98.4 ± 0.2%. There was no effect of sequencing platform on coverage (ANOVA; *F* = 1.59, *P* > .05), which averaged 83%. 7576 singletons were removed from the dataset, leaving 8807 ASVs for downstream analysis.

### Environmental conditions of rivers

In the TRB, the Duck River had higher daily temperature (26.1 ± 2.8 °C; *F* = 15.35, *P* < .001), conductivity (250.8 ± 109.1 μS cm^−1^; *F* = 11.22, *P* < .001), and SRP (93.8 ± 8.2 μg L^−1^; *F* = 139.6, *P* < .001) than the Paint Rock (24.7 ± 2.6 °C, 303.9 ± 31.2 S cm^−1^, and 11.5 ± 1.7 μg L^−1^) (Fig. S1A–C). There were significant differences by sampling week for temperature (*F* = 82.40, *P* < .001) and conductivity (*F* = 59.46, *P* < .001), and the river × week interaction was significant for temperature (*F* = 1.923, *P* < .05), conductivity (*F* = 59.46, *P* < .001), and SRP (*F* = 2.687, *P* < .05). NH_4_^+^ did not differ between the Duck and Paint Rock (22.2 ± 7.1 μg L^−1^ and 22.5 ± 10.1 μg L^−1^, respectively; *F* = 0.291, *P* > .05; Fig. S1D) but did differ by week (*F* = 98.47, *P* < .001) with a significant river × week interaction (*F* = 7.403, *P* < .001).

The Sipsey (26.9 ± 3.4 °C) and Cahaba (26.7 ± 3.0 °C) rivers in the MRB had similar daily water temperatures; temperatures decreased by week (*F* = 285.5, *P* < .001) with a river × week interaction (*F* = 2.057, *P* < .05; Fig. S1E). Conductivity in the Cahaba (289.0 ± 48.9 μS cm^−1^) was twice that of the Sipsey (131.1 ± 33.4 μS cm^−1^, Fig. S1F), and the Cahaba had higher SRP (12.4 ± 5.2 μg L^−1^ vs. 10.5 ± 3.8 μg L^−1^ in the Sipsey; Fig. S1F) and NH_4_^+^ (30.2 ± 12.8 μg L^−1^ vs. 21.4 ± 7.2 μg L^−1^; Fig. S1H; *F* = 139.6–161.5, *P* < .001). There were significant river × week interactions for all water parameters (*F* = 15.64–458.9, *P* < .001).

### Bacterial community composition of mussels, sediment, and seston

**Figure 1:**
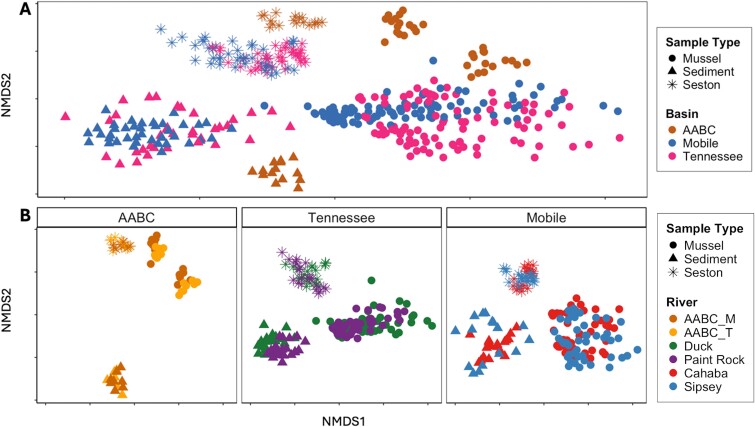
NMDS plots based on Bray–Curtis dissimilarity scores of bacterial communities between sample types (mussel gut, sediment, seston) separated by river basin (A) and river of sample placement or collection (B). Basin and river are separated by color and sample type is differentiated by shape. Mussel gut samples are from hatchery-propagated *Lampsilis ovata* placed in the Tennessee River basin and *L. ornata* placed in the Mobile River basin, as well as mussels sampled directly from the hatchery (AABC). AABC_M and AABC_T refer to *L. ornata* and *L. ovata* mussels, respectively, that were directly sampled from the AABC as the baseline for mussels placed into the Mobile or Tennessee River basins. For clarity of sample separation, non-significant outliers (eight sediment samples) were removed from the ordination. Sediment, seston, and mussel gut samples all grouped distinctly within all sampling areas (AABC, Mobile River basin, and Tennessee River basin).

Bacterial community composition varied with sample type (sediment, seston, mussel; *R*^2^ = 0.17, *F* = 46.63, *P* < .001) and source/basin (AABC, TRB, MRB; *R*^2^ = 0.07, *F* = 10.49, *P* < .001; Fig. 1A and B), with some variation explained by the sample type × source interaction (*R*^2^ = 0.12, *F* = 7.50, *P* < .001). For AABC samples, differences between sediment and seston explained the most variation in community composition (*R*^2^ = 0.40, *P*-adj < .01), followed by differences between sediment and mussels (*R*^2^ = 0.31, *P*-adj < .01), and seston and mussels (*R*^2^ = 0.22, *P*-adj < .01). The gut bacterial community of mussels collected at Week 0 and Week 16 directly from the AABC differed significantly (*R*^2^ = 0.54, *F* = 35.28, *P* < .001). In the TRB, the most variation in community composition was explained by differences between sediment and seston (*R*^2^ = 0.24, *P*-adj < .01), followed by mussels and seston (*R*^2^ = 0.20, *P*-adj < .01). For MRB samples, mussel gut communities and seston were the most different (*R*^2^ = 0.22, *P*-adj < .01), followed by sediment and seston (*R*^2^ = 0.21, *P*-adj < .01). For both basins, the least amount of variation was explained between mussel and sediment bacterial communities (*R*^2^ = 0.14–0.15, *P*-adj < .01).

Seston bacterial communities differed between the Duck and Paint Rock rivers in the TRB (*R*^2^ = 0.12, *F* = 15.89, *P* < .001) and by week (*R*^2^ = 0.42, *P* < .001), with a river × week interaction (*R*^2^ = 0.25, *F* = 9.33, *P* < .001). Dissimilarity of seston communities was correlated with differences in temperature (Mantel’s correlation (*r*) = 0.711, *P* < .001) and SRP (*r* = 0.311, *P* < .001), but not conductivity (*r* = −0.014, *P* > .05) or NH_4_^+^ (*r* = 0.090, *P* > .05). Sediment communities also differed between the Duck and Paint Rock (*R*^2^ = 0.12, *F* = 6.02, *P* < .001) and by week (*R*^2^ = 0.18, *F* = 1.49, *P* < .001), with a significant interaction (*R*^2^ = 0.16, *F* = 1.36, *P* < .001). Dissimilarity of sediment bacterial communities was also correlated with differences in temperature (*r* = 0.169, *P* < .05) and SRP (*r* = 0.465, *P* < .001), but not conductivity or NH_4_^+^ (*r* = −0.044–0.100, *P* > .05). Bacterial species richness in seston or sediment from either river was unrelated to sampling week (adj-*R*^2^ = 0.04, *P* = .11 and adj-*R*^2^ = 0.01, *P* = .27, respectively; Fig. S2A).

In the MRB, seston bacterial community composition differed between the Cahaba and Sipsey (*R*^2^ = 0.34, *F* = 29.68, *P* < .001), by week (*R*^2^ = 0.21, *F* = 6.23, *P* < .001), with a river × week interaction (*R*^2^ = 0.25, *F* = 5.52, *P* < .001). Dissimilarity in the seston community correlated with differences in temperature (*r* = 0.236, *P* < .001), conductivity (*r* = 0.654, *P* < .001), SRP (*r* = 0.321, *P* < .001), and NH_4_^+^ (*r* = 0.172, *P* < .001). The sediment bacterial community also differed between these rivers (*R*^2^ = 0.22, *F* = 8.07, *P* < .001), by week (*R*^2^ = 0.13, *F* = 1.62, *P* < .001), with a significant interaction (*R*^2^ = 0.19, *F* = 1.71, *P* < .001). Sediment community dissimilarity was correlated with conductivity (*r* = 0.416, *P* < .001), but not temperature, SRP, or NH_4_^+^ (*r* = −0.009–0.009, *P* > .05). Bacterial species richness in seston and sediment from rivers in the MRB was unrelated to sampling week (adj-*R*^2^ = 0.01, *P* = .28 and adj-*R*^2^ = 0.02, *P* = .19, respectively; Fig. S2B).

### Changes in the gut microbiome of hatchery-propagated mussels after placement in rivers

**Figure 2:**
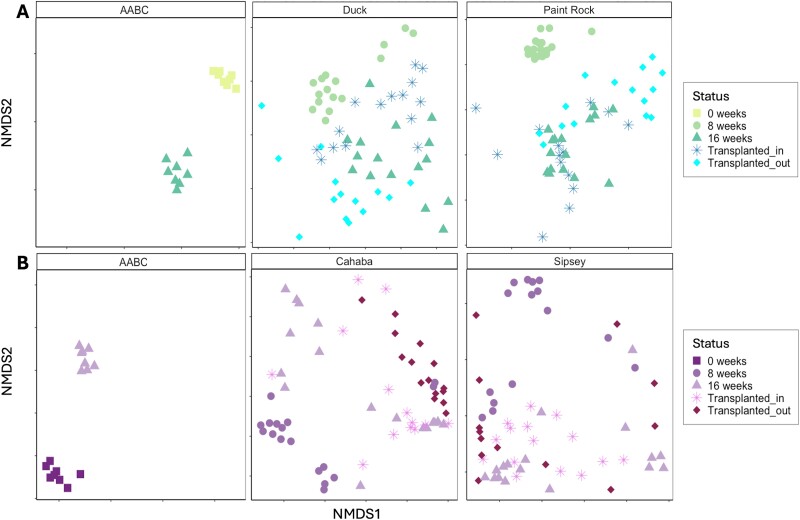
NMDS plots based on bray–Curtis dissimilarity scores of bacterial communities and separated by the status of each group for gut samples from hatchery-propagated mussels placed into rivers in the Tennessee River basin (*Lampsilis ovata*; A) and the Mobile River basin (*L. ornata*; B). Plots are separated based on the river that each mussel was placed into (Duck, Paint Rock, Cahaba, Sipsey) with mussels that remained in the hatchery designated AABC. Status refers to the duration that each mussel remained in the river. Reciprocally transplanted mussels are included in plots of their original river, from 0 to 8 weeks (i.e. transplanted_out), and their final river, from 8 to 16 weeks (i.e. transplanted_in). Separation of *L. ovata* and *L. ornata* samples occurred between Week 0, Week 8, and Week 16 gut microbiomes, with transplanted mussel gut microbiomes being more similar to Week 16 mussel samples from the river to which they were transplanted into (i.e. Transplanted_in).

Gut bacterial communities of *L. ovata* differed based on the TRB river they were initially placed in (*R*^2^ = 0.25, *F* = 18.25, *P* < .001) and by time (*R*^2^ = 0.20, *F* = 14.63, *P* < .001), with a small river × time interaction (*R*^2^ = 0.05, *F* = 7.88, *P* < .001; Fig. 2A). The greatest variation was between *L. ovata* at the AABC and those collected after placement in the Duck (*R*^2^ = 0.21) or Paint Rock (*R*^2^ = 0.27) rivers. Temporal differences in the gut communities of *L. ovata* were most distinct between weeks 0 and 8 (*R*^2^ = 0.30, *P*-adj < .01), followed by weeks 0 and 16 (*R*^2^ = 0.23, *P*-adj < .01), and weeks 8 and 16 (*R*^2^ = 0.13, *P*-adj < .01). Bacterial communities of *L. ornata* placed in the MRB also differed by river (*R*^2^ = 0.22, *F* = 15.7, *P* < .01) and collection time (*R*^2^ = 0.19, *F* = 13.0, *P* < .01; Fig. 2B), with a river × time interaction (*R*^2^ = 0.05, *F* = 6.50, *P* < .01). The greatest variation in gut communities of *L. ornata* was between mussels sampled from the AABC and those collected after placement in the Sipsey (*R*^2^ = 0.28, *F* = 15.73, *P*-adj < .01), followed by those from the AABC and the Cahaba (*R*^2^ = 0.20, *F* = 13.00, *P*-adj < .01), and mussels from the Cahaba and the Sipsey (*R*^2^ = 0.10, *F* = 6.50, *P*-adj < .01). Gut bacterial communities of *L. ornata* at Week 0 differed from those at weeks 8 (*R*^2^ = 0.31, *P*-adj < .01) and 16 (*R*^2^ = 0.20, *P*-adj < .01). Differences between weeks 8 and 16 explained less variance (*R*^2^ = 0.09, *P*-adj < .01).

**Figure 3:**
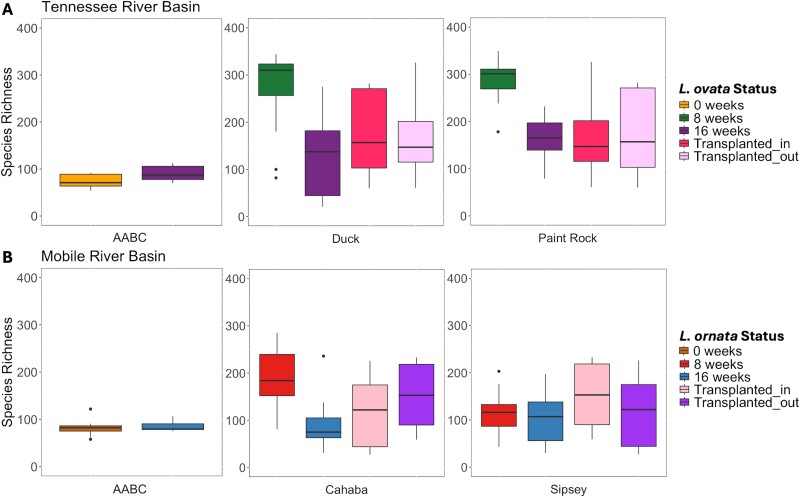
Species richness (S_obs_) of the gut bacterial community of hatchery-propagated mussels *Lampsilis ovata* (A) and *L. ornata* (B) placed into rivers in the Tennessee River basin and Mobile River basin. Plots are separated based on the river that each mussel was placed into (Duck, Paint Rock, Cahaba, Sipsey), with mussels that remained in the hatchery designated AABC. Status refers to the duration that each mussel remained in that river. Reciprocally transplanted mussels are included in plots of their original river, from 0 to 8 weeks (i.e. Transplanted_out), and their final river, from 8 to 16 weeks (i.e. Transplanted_in). Boxes show the interquartile ranges/distributions of values measured in each metric, with the black solid line representing the median value from the sample type. Vertical lines represent the highest and lowest values associated with each grouping variable. Dots represent outliers from each group.

Species richness of the mussel gut microbiome was explained by collection week (*X*^2^ = 41.96, *P* < .001; Fig. 3A) but not river (*X*^2^ = 3.61, *P* > .05). Microbiome species richness of *L. ovata* after 8 weeks in the Duck or Paint Rock (mean ± standard error 279 ± 11) was greater than for mussels from the AABC at Week 0 (73 ± 5; *P*-adj < .001) and those collected from rivers at Week 16 (135 ± 9; *P*-adj < .001). Similarly, species richness of the *L. ornata* gut microbiome was predicted by collection week (*X*^2^ = 7.15, *P* < .05; Fig. 3B), but not river (*X*^2^ = 2.45, *P* > .05). *L. ornata* collected from rivers at Week 8 had a richer gut bacterial community than those collected at Week 16 (mean S_obs_ of 153 ± 11 and 94 ± 7; adj-*P* < .05).

Eleven bacterial phyla/subphyla accounted for >85% of the sequences recovered from *L. ovata* (Fig. S3): *Firmicutes* (22.4% of sequences), *Alphaproteobacteria* (16.1%), *Planctomycetes* (11.1%), *Fusobacteria* (9.4%), *Cyanobacteria* (5.3%), *Gammaproteobacteria* (4.5%), *Verrucomicrobia* (4.4%), *Betaproteobacteria* (4.2%), *Actinobacteria* (3.9%), *Acidobacteria* (2.9%), and *Bacteroidetes* (1.7%). 10.3% of sequences from *L. ovata* could not be classified further than bacteria. The proportion of each taxon differed by collection week (MANOVA; *F* = 4.12–246.96, *P* < .001–.05) for all phyla other than *Bacteroidetes*, and by site (*F* = 4.39–78.86, *P* < .001–.05) for phyla other than *Actinobacteria* and *Firmicutes*. After 8 weeks in the Duck and Paint Rock rivers, proportions of *Cyanobacteria* and unclassified taxa in the *L. ovata* gut community were reduced compared to initial specimens from the AABC (TukeyHSD; *P*-adj < .001). Proportions of other bacterial phyla increased after 8 weeks in the rivers (*P*-adj < .001–.05). The gut microbiome of *L. ovata* at the AABC also changed over the experimental period, with reductions in proportions of *Cyanobacteria* and unclassified sequences (*F* = 38.22, *P* < .001 and *F* = 102.20, *P* < .001) and increases in proportions of *Firmicutes* (*F* = 82.23, *P* < .001), *Planctomycetes* (*F* = 5.85, *P* < .05), *Verrucomicrobia* (*F* = 20.88, *P* < .001), and *Betaproteobacteria* (*F* = 15.34, *P* < .01). *L. ovata* in the Duck and Paint Rock rivers showed further changes in their gut community between weeks 8 and 16, with decreases in proportions of *Planctomycetes*, *Gammaproteobacteria*, *Verrucomicrobia*, and *Betaproteobacteria* for mussels in both rivers (*F* = 8.65–34.66, *P* < .001–.05 and *F* = 44.30–125.70, *P* < .001–.05, respectively), and reductions in proportions of *Cyanobacteria* (*F* = 4.76, *P* < .05) and *Actinobacteria* (*F* = 23.05, *P* < .001) for mussels in the Duck River, and *Alphaproteobacteria* (*F* = 22.01, *P* < .001) and *Acidobacteria* (*F* = 26.34, *P* < .001) for mussels in the Paint Rock. The proportion of *Alphaproteobacteria* in the gut microbiome increased for *L. ovata* placed in the Duck River (*F* = 14.79, *P* < .001), while proportions of *Fusobacteria* and *Firmicutes* increased for mussels in the Paint Rock (*F* = 36.88, *P* < .001 and *F* = 4.76, *P* < .05). The gut microbiome of *L. ovata* placed in the Paint Rock had greater proportions of *Firmicutes* (*F* = 6.17, *P* < .05), *Cyanobacteria* (*F* = 19.96, *P* < .001), and *Gammaproteobacteria* (*F* = 17.61, *P* < .001) compared to mussels placed in the Duck River.

For *L. ornata*, 10 bacterial phyla accounted for >90% of sequences recovered from the gut community (Fig. S4): *Firmicutes* (27.7% of sequences), *Betaproteobacteria* (19.1%), *Planctomycetes* (11.0%), *Alphaproteobacteria* (10.0%), *Cyanobacteria* (9.5%), *Fusobacteria* (4.1%), *Gammaproteobacteria* (2.9%), *Verrucomicrobia* (2.7%), *Actinobacteria* (1.7%), and *Bacteroidetes* (1.1%), with 7.7% of sequences being unclassified. Proportions of most phyla differed by collection week (*F* = 3.59–273.67, *P* < .001–.05), exceptions being *Bacteroidetes* and *Gammaproteobacteria*. Proportions of *Betaproteobacteria*, *Cyanobacteria*, *Fusobacteria*, *Gammaproteobacteria*, and *Actinobacteria* in the *L. ornata* gut microbiome differed based on site (*F* = 6.26–48.87, *P* < .001–.01). As with *L. ovata*, *L. ornata* sampled initially from the AABC contained higher proportions of *Cyanobacteria* and unclassified taxa in their gut microbiome, which declined after 8 weeks in the Cahaba or Sipsey (*P*-adj < .001), or after 16 weeks in the AABC (*P*-adj < .001). Proportions of *Firmicutes*, *Planctomycetes*, *Alphaproteobacteria*, *Fusobacteria*, *Verrucomicrobia*, and *Bacteroidetes* were also greater in Week 16 AABC *L. ornata* compared to Week 0 (*F* = 4.97–79.31, *P* < .001–.05). For *L. ornata* placed in the Cahaba and Sipsey rivers, proportions of *Planctomycetes* and *Fusobacteria* in the gut microbiome decreased throughout the study (*F* = 4.44–11.20, *P* < .01–.05 and *F* = 30.54–76.21, *P* < .001, respectively), as did proportions of *Verrucomicrobia* (*F* = 16.75, *P* < .001) and *Actinobacteria* (*F* = 15.77, *P* < .001) for mussels in the Cahaba River, and *Cyanobacteria* (*F* = 34.77, *P* < .001) and *Bacteroidetes* (*F* = 7.94, *P* < .01) for mussels in the Sipsey. The gut microbiome of *L. ornata* had higher proportions of *Cyanobacteria* for mussels placed in the Cahaba River (*F* = 9.11, *P* < .01) and *Fusobacteria* for mussels placed in the Sipsey (*F* = 10.06, *P* < .01).

**Figure 4:**
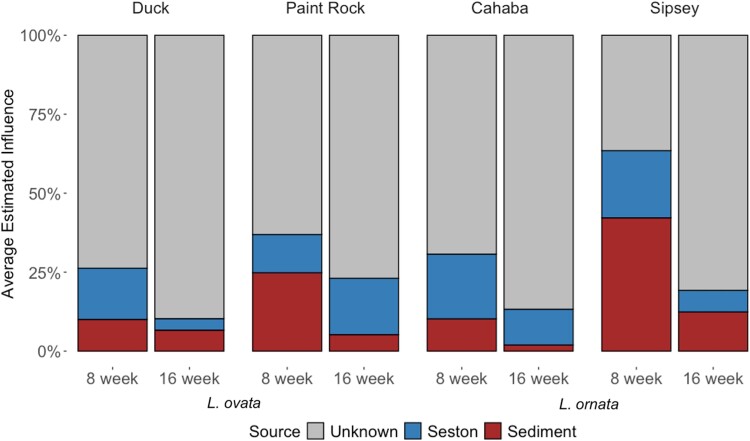
FEAST analysis depicting the influence (%) of the bacterial communities from seston and sediment, from 0 to 8 weeks (8 week) and 8 to 16 weeks (16 week), and unknown sources on the gut microbiome of hatchery-propagated *Lampsilis ovata* and *L. ornata* mussels placed into the Duck and Paint Rock rivers of the Tennessee River basin (*L. ovata*) and the Cahaba and Sipsey rivers of the Mobile River basin (*L. ornata*). Each bar represents the average estimated influence of each source of bacteria to the gut microbiome of mussels (sinks) within each river. Seston and sediment sources are separated by color and based on sampling weeks, and unknown sources are colored in gray.

The gut community of *L. ovata* was more influenced by sediment bacterial communities at 0 or 8 weeks than after Week 8 (MANOVA; *F* = 9.47, *P* < .01; Fig. 4), a similar pattern that was suggested, but not significant, for *L. ornata* (*F* = 2.66, *P* = .13). The gut microbiome of *L. ornata* was more influenced by seston bacterial communities collected up to 8 weeks compared to those collected after Week 8 (*F* = 8.17, *P* < .05; Fig. 4). Gut communities of *L. ovata* and *L. ornata* at weeks 8 and 16 were most influenced by unknown sources of bacteria, and this influence was greater after 16 weeks in their respective rivers (*F* = 11.16, *P* < .001 and *F* = 8.79, *P* < .05, respectively). The influence of bacterial communities in sediment, seston, or unknown sources on the gut bacterial communities of *L. ovata* or *L. ornata* did not differ significantly between rivers.

### Impacts of transplants between rivers on the gut microbiome of freshwater mussels

The gut microbiome of *L. ovata* differed between mussels that remained in the same river for 16 weeks and those that were reciprocally transplanted between the Duck and Paint Rock rivers (PERMANOVA; *R*^2^ = 0.17, *F* = 4.06, *P* < .001; Fig. 2A). *L. ovata* transplanted from the Duck to the Paint Rock (D-PR transplants) had gut communities that differed from mussels that remained in the Duck River for 16 weeks (*R*^2^ = 0.15; *P*-adj < .01), but not from mussels that were in the Paint Rock for the whole study (*R*^2^ = 0.05, *P*-adj = .51). Gut communities of *L. ovata* transplanted from the Paint Rock to the Duck (PR-D transplants) differed from those that remained in the Paint Rock or Duck River for 16 weeks (*R*^2^ = 0.21 and *R*^2^ = 0.12, respectively, *P*-adj < .01). The same pattern was seen in the MRB, where reciprocally transplanted *L. ornata* had gut microbiomes that differed from those that had remained in the Cahaba or Sipsey for all 16 weeks (*R*^2^ = 0.12, *F* = 4.26, *P* < .001; Fig. 2B). Gut microbiomes of *L. ornata* that were transplanted from the Cahaba to the Sipsey (C-S transplants) differed from those that remained in the Cahaba (*R*^2^ = 0.16, *P*-adj < .01), but not from mussels that were placed in the Sipsey initially and remained there (*R*^2^ = 0.06, *P*-adj = .54). Pairwise comparisons revealed no differences between the gut microbiomes of *L. ornata* transplanted from the Sipsey to the Cahaba (S-C transplants) and *L. ornata* that were in the Cahaba (*R*^2^ = 0.06, *P*-adj = 0.73) or Sipsey (*R*^2^ = 0.09, *P*-adj = 0.13) for the whole study.

The species richness of the bacterial gut microbiome of mussels that were transplanted between rivers was similar to that of mussels that remained in the receiving river for the entire experiment. For *L. ovata*, gut bacterial species richness of PR-D transplants was less than that of Week 8 *L. ovata* in the Paint Rock (*P*-adj < .05). There were no significant differences in bacterial species richness of transplanted *L. ornata* compared to mussels in the Cahaba or Sipsey rivers at Week 8 (*P*-adj > .05).

**Figure 5:**
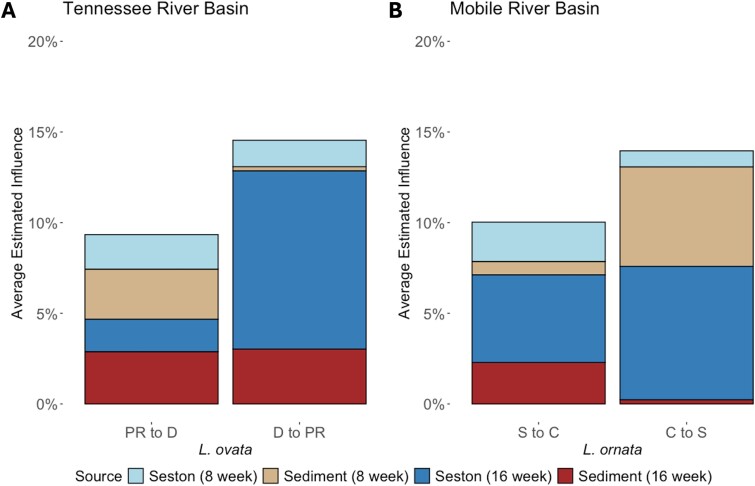
FEAST analysis depicting the influence (%) of the bacterial communities from seston and sediment, from 0 to 8 weeks (8 week) and 8 to 16 weeks (16 week), on the gut microbiome of (A) *Lampsilis ovata* and (B) *L. ornata* mussels that were reciprocally transplanted between the Paint Rock (PR) and Duck (D) rivers of the Tennessee River basin, and between the Sipsey (S) and Cahaba (C) rivers of the Mobile River basin. Each bar represents the mean estimated influence of bacteria from each source to the gut microbiome of mussels (sinks) within each river. Seston and sediment sources are separated by color and based on sampling weeks. Unknown sources accounted for the remaining (average 88%) of influence on the community.

Bacteria from unknown sources represented 88% of the influence on the gut microbiomes of transplanted mussels. Of sources that were identified, the gut communities of D-PR transplanted *L. ovata* were most influenced by seston bacterial communities in the receiving Paint Rock River (9.83%; *F* = 14.40, *P* < .01; *P*-adj < .05), whereas no source of bacteria contributed more than another to gut microbiomes of PR-D transplanted *L. ovata* (*F* = 0.23, *P* = .88; Fig. 5A)*.* For *L. ornata*, gut communities of S-C transplants were most influenced by seston in the receiving Cahaba River (4.83%; *F* = 1.56, *P* = .27), and C-S transplants also had gut microbiomes that were influenced by bacterial communities on seston in the river (the Sipsey) that received them (*F* = 4.24, *P* < .05; 7.36%; Fig. 5B). C-S transplanted *L. ornata* were also influenced by bacteria in sediment at their initial site in the Cahaba (5.45%).

Patterns in transplanted mussels were apparent in the ASVs that were commonly present in their gut bacterial communities. In the Duck River, ASV6 (identified as Bacilli) and ASV2 (also Bacilli) were the two most frequently detected bacteria in *L. ovata*, found in 93.8% and 87.5%, respectively, of *L. ovata* at 16 weeks (Table S1). These ASVs were also more prevalent in PR-D transplanted *L. ovata* (86.7% and 93.3% of mussels, respectively) compared to mussels that remained in the Paint Rock for 16 weeks (75.0% and 43.8%). The gut microbiomes of D-PR transplants were less likely to contain ASV6 (42.9% mussels) and ASV2 (50.0%) compared to those that remained in the Paint Rock. ASV3 (*Cetobacterium*) and ASV29 (*Rhizobiales*) were found in 100% of *L. ovata* that remained in the Paint Rock for 16 weeks, and these ASVs were more frequently detected in D-PR transplants (92.9% mussels for ASV3, 64.3% for ASV29) than in mussels that remained in the Duck River (68.8% ASV3, 0% ASV29). PR-D transplants were less likely to contain ASV3 in their gut microbiome (60.0% mussels) than those that stayed in the Paint Rock (100%), and ASV 29 was not commonly detected in gut communities of PR-D transplants.

For *L. ornata*, ASV10 (*Fimbriiglobus*) and ASV26 (unidentified *Cyanobacteria*) were found in 94.1% and 82.4%, respectively, of mussels that remained in the Cahaba for 16 weeks, and these were at higher frequencies (76.9% and 69.2%) in S-C transplants compared to mussels that remained in the Sipsey or in C-S transplants, where they were not a frequently detected ASV (Table S1). C-S transplants often contained ASV66 (*Firmicutes*; 93.3% of mussels) and ASV51 (*Romboutsia sedimentorum*; 66.7% of mussels), and these were found in 64.7% and 82.4%, respectively, of *L. ornata* that remained in the Sipsey for all 16 weeks. Neither of these ASVs were among the most frequently identified ASVs in the gut bacterial community of *L. ornata* that were in the Cahaba River for 16 weeks, although ASV51 was found in 53.8% of S-C transplants (Table S1).

## Discussion

We used hatchery-propagated animals to determine the influence of different rivers on the gut microbiome of two species of freshwater mussels, *L. ovata* and *L. ornata*. Our results demonstrate that when mussels are introduced into new environments, bacterial populations rapidly colonize their gut, increasing the diversity of their gut bacterial community, and transplants between rivers cause additional changes in their gut microbiome. Further, length of time in rivers exerted a greater influence on the gut microbiome than environmental parameters or environmental bacterial communities. This study represents one of the first to explore structural changes in the gut microbiome of captivity-raised mussels placed into the wild, providing insights for conservation efforts such as species reintroduction and translocation initiatives.

After just 8 weeks in each river, the gut microbiome of hatchery-propagated mussels increased in species richness, while mussels remaining in the hatchery maintained a lower richness. Lower gut diversity of hatchery-raised mussels compared to wild mussels has been shown for *Villosa nebulosa* (now classified as *Cambarunio nebulosus*) [27], and this could reflect dietary differences between mussels in a natural river and those fed a consistent diet in a hatchery facility. Bacterial species richness subsequently decreased for mussels that remained in the same river for the study duration, suggesting that the gut microbiome may have begun to stabilize with “core” gut bacterial populations, rather than potentially transient species that were present at Week 8. Neither gut bacterial community composition nor species richness correlated with specific environmental parameters, suggesting that host development, food sources, or other parameters drove these patterns.

The gut microbiomes of mussels were distinct from bacterial communities of seston and sediment and differed between rivers in the same basin, consistent with previous findings showing site specificity of the mussel gut microbiome and its distinction from bacterial communities in the environment [22, 25, 26]. While we did not identify an environmental variable that exerted a greater influence than time on gut bacterial community structure, reciprocal transplants indicated a role of site in microbiome composition. In both river basins, the gut microbiomes of transplanted mussels resembled those of Week 16 mussels from the same final river but differed from those in the river they were transplanted from. Moreover, the gut species richness of transplanted mussels was similar to that of mussels that were placed in that river initially, suggesting that the gut microbiome responds to a changing environment even as it stabilizes.

We used young, hatchery-propagated mussels, and as mussels mature, their feeding shifts from pedal-feeding on sediment to filter-feeding seston in the water column [30, 52, 53]. FEAST analyses did not indicate that sediment and seston bacterial communities significantly influenced the gut microbiome of these mussels, although there was a suggestion that the gut microbiome of Week 8 mussels was more influenced by sediment, while later samples were more influenced by seston. Bacterial communities on seston and sediment have been found to have minimal influence on the gut microbiome of adult mussels compared to the influence of coexisting species [26]. Thus, the unidentified sources (which averaged an estimated influence of 88%) in our FEAST analysis likely represent the influence of co-occurring mussels within the same silos.

Hatchery-propagated mussels were fed a shellfish diet consisting of microalgae, which likely contributed to sequences classified as *Cyanobacteria* and unidentified phyla in the mussels’ guts. The proportion of *Cyanobacteria* was reduced in the gut microbiome of mussels after they spent 8 weeks in natural rivers, and the proportion of sequences classified as *Firmicutes*, *Alphaproteobacteria*, *Betaproteobacteria*, *Planctomycetes*, and *Fusobacteria* increased, consistent with phyla observed in the gut microbiomes of adult mussels in natural systems [13, 22–26]. Mussels that remained in the AABC exhibited more similar gut bacterial communities to those placed in natural rivers as the study progressed, suggesting that these phyla may become more prevalent as mussels mature, regardless of location or food source. *Firmicutes* may represent a core phylum for *Lampsilis* species, as they occupied a substantial fraction of the gut community in all Week-16 mussels. *Romboutsia sedimentorium* (*Firmicutes*) has been previously identified as part of the core gut microbiome of native and invasive freshwater bivalves [22, 23, 26, 54] and was found at high frequencies in *L. ornata* that were in natural rivers for 16 weeks, whether transplanted at Week 8 or not, suggesting a relationship between this bacterium and freshwater mussels.

Growth and survival of freshwater mussels are influenced by hydrology [28, 55], contaminants [56, 57], temperature [29, 30], and substratum [58]. Shifts in the virome have been detected in mussels introduced into natural environments from hatcheries [59], but few studies have analyzed the impact of new environments on the bacterial gut microbiome of mussels. While no specific environmental variables were identified as influencing the gut microbiome in this study, the role of site overall was highlighted by reciprocal transplants and underscores the importance of prevailing environmental conditions. As hatchery-propagated mussels acclimated in natural rivers, their gut microbiome shifted from one dominated by their hatchery food source, to one that was more representative of that of adult, wild-collected mussels. These findings contribute to our understanding of mussel gut microbiome development and highlight the need for conservation strategies to consider microbiome dynamics to enhance restoration success [60, 61]. This may be especially important for species reintroduction initiatives, where animals raised in captivity may have different microbiomes than wild populations. Our findings suggest that approaches such as acclimation under natural conditions (e.g. in recipient river water for freshwater mussels) may be required to shift the gut microbiome prior to release into the wild, especially if gut microbiome composition relates to competitive success. Exploration of the multifaceted relationships between mussels, their environment, and their gut microbiome holds promise for informing management approaches aimed at increasing the health and resilience of these ecologically important organisms.

## Supplementary Material

Vaughn_et_al_ISME_supplemental_ycae125

## Data Availability

The datasets generated during/and analyzed during the current study are available in the NCBI Sequence Reads Archive under BioProject ID PRJNA1125911.
