# Supplementary material for: Introduction into natural environments shifts the gut microbiome of captivity-raised filter-feeding bivalves
Source: ISME Commun. 2024 Oct 23;4(1):ycae125. doi: 10.1093/ismeco/ycae125 (PMC11538807; doi:10.1093/ismeco/ycae125)
Supplement: Vaughn_et_al_ISME_supplemental_ycae125 [file vaughn_et_al_isme_supplemental_ycae125.pdf]

Introduction into natural environments shifts the gut microbiome of captivity-raised filter-feeding bivalves

Short Title: Shifts in freshwater mussel microbiomes

Stephanie N. Vaughn, Garrett W. Hopper, Irene Sánchez González, Jamie R. Bucholz, Ryan C. Garrick, Jeffrey D. Lozier, Paul D. Johnson, Carla L. Atkinson, and Colin R. Jackson

Supplemental Figures and Tables

Figure S1. Physicochemical parameters in four rivers (Duck, Paint Rock, Cahaba, Sipsey) in the Tennessee (A-D) and Mobile (E-F) River Basins. Temperature (˚C; A, E) and conductivity (μS cm^{-1}; B, F) were measured every hour and averaged daily. Soluble reactive phosphorus (SRP, μg L^{-1}; C, G) and NH_4^+ (μg L^{-1}; D, H) were measured every 2-3 weeks (mean of three replicates; error bars represent standard deviation from the mean). Measurements taken over a total of 112 days/16 weeks from May-August 2022 for the Tennessee River Basin and June-October 2022 for the Mobile River Basin.

Figure S2. Bacterial species richness (S_{obs}) of seston and sediment bacterial communities collected from the Duck and Paint Rock rivers of the Tennessee River Basin (A) and the Cahaba and Sipsey rivers of the Mobile River Basin (B) over 16 weeks. Each point is representative of one sample with a total of six samples per week (n=3 replicates per river) and separated by color based on collection river.

Figure S3. Major bacterial phyla, as determined from percentage of 16S rRNA gene sequences recovered, detected in the gut bacterial communities of hatchery-propagated *Lampsilis ovata* from the Alabama Aquatic Biodiversity Center (AABC) and placed in the Duck and Paint Rock rivers of the Tennessee River Basin. Each bar represents one individual and grouped based on mussel age/time in river (i.e. 0 weeks, 8 weeks, or 16 weeks).

Figure S4. Major bacterial phyla, as determined from percentage of 16S rRNA gene sequences recovered, detected in the gut bacterial communities of hatchery-propagated *Lampsilis ornata* from the Alabama Aquatic Biodiversity Center (AABC) and placed in the Cahaba and Sipsey rivers of the Mobile River Basin. Each bar represents one individual and grouped based on mussel age/time in river (i.e. 0 weeks, 8 weeks, or 16 weeks).

Table S1. The core gut microbiome of 16 week and reciprocally transplanted *Lampsilis ovata* and *L. ornata* placed into the Duck (D) and Paint Rock (PR) rivers of the Tennessee River Basin and the Cahaba (C) and Sipsey (S) rivers of the Mobile River Basin. ASVs listed comprised >1% of all sequences detected in the gut of each mussel and are ordered based on relative abundance within each group. Frequency was determined from the number of individual gut samples within each group that yielded that ASV. Identifications for each ASV were made to the phylum level followed by the corresponding finest classified taxonomy possible in parentheses.

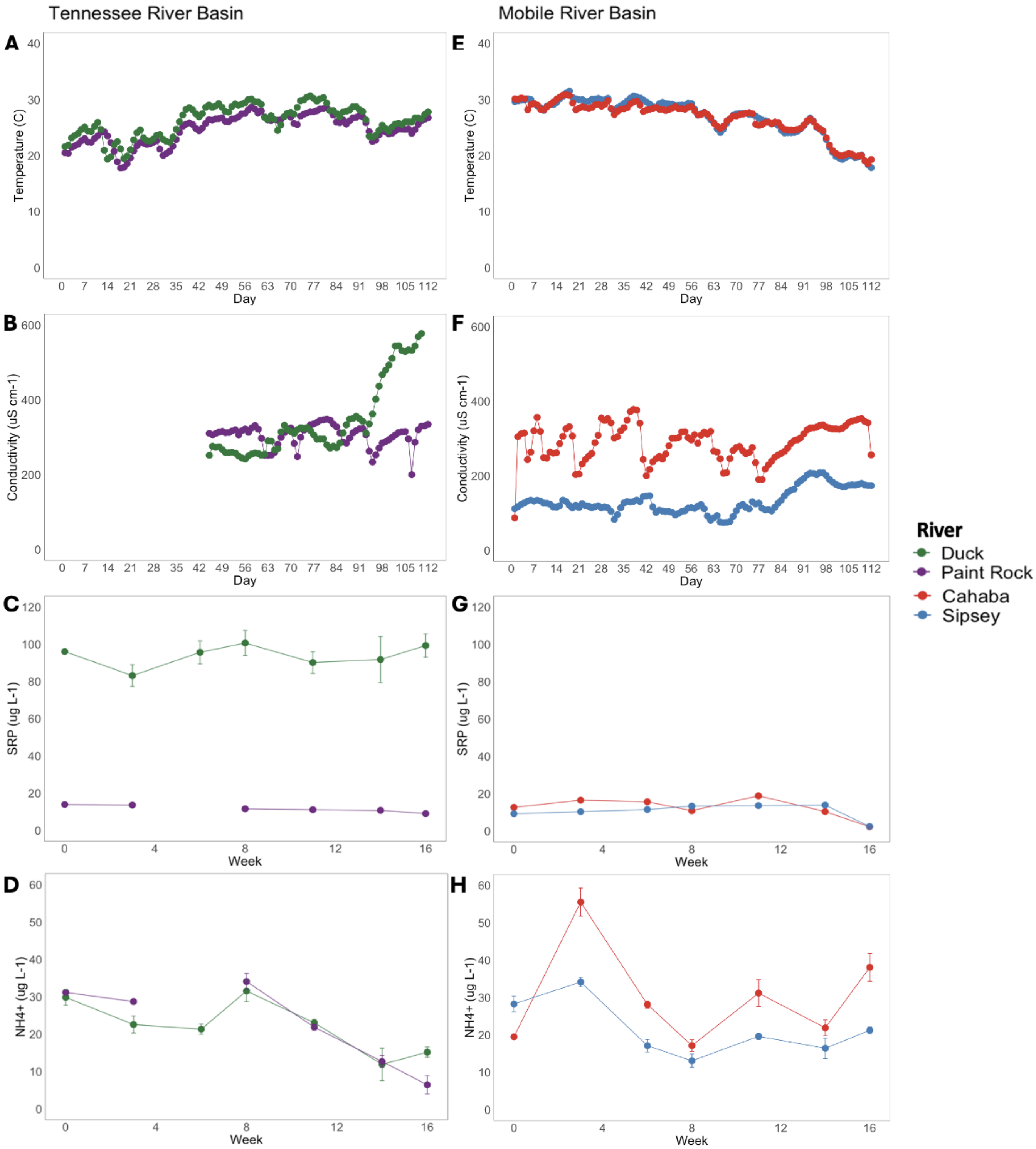

Figure S1. Physicochemical parameters in four rivers (Duck, Paint Rock, Cahaba, Sipsey) in the Tennessee (A-D) and Mobile (E-F) River Basins. Temperature (˚C; A, E) and conductivity (µS cm^{-1}; B, F) were measured every hour and averaged daily. Soluble reactive phosphorus (SRP, µg L^{-1}; C, G) and NH_4^+ (µg L^{-1}; D, H) were measured every 2-3 weeks (mean of three replicates; error bars represent standard deviation from the mean). Measurements taken over a total of 112 days/16 weeks from May-August 2022 for the Tennessee River Basin and June-October 2022 for the Mobile River Basin.

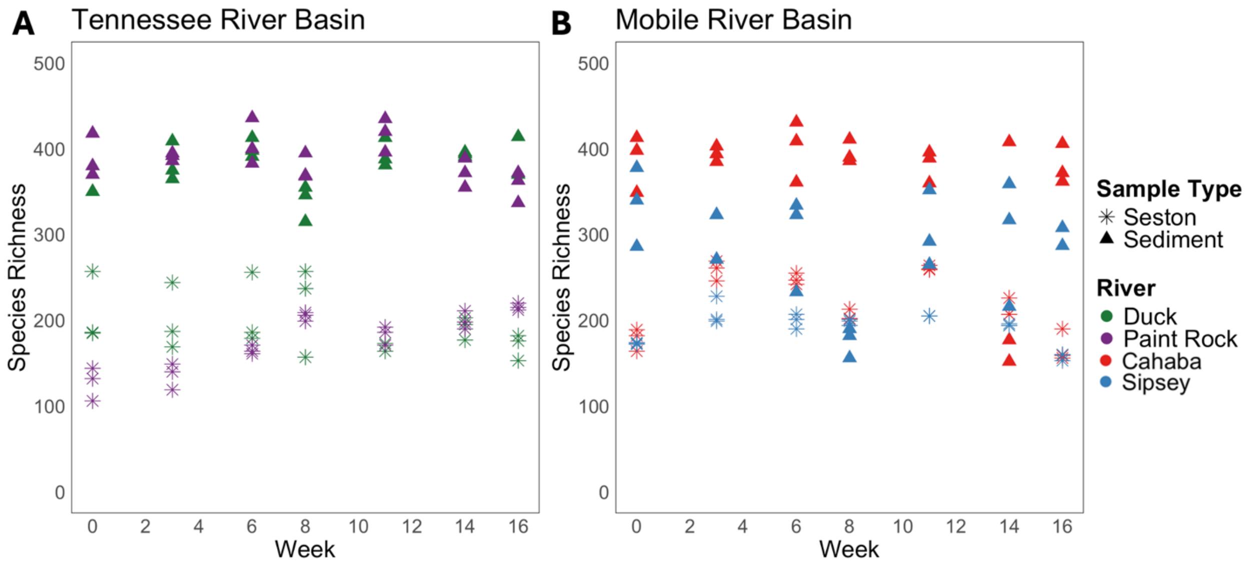

Figure S2. Bacterial species richness (S_{obs}) of seston and sediment bacterial communities collected from the Duck and Paint Rock rivers of the Tennessee River Basin (A) and the Cahaba and Sipsey rivers of the Mobile River Basin (B) over 16 weeks. Each point is representative of one sample with a total of six samples per week (n=3 replicates per river) and separated by color based on collection river.

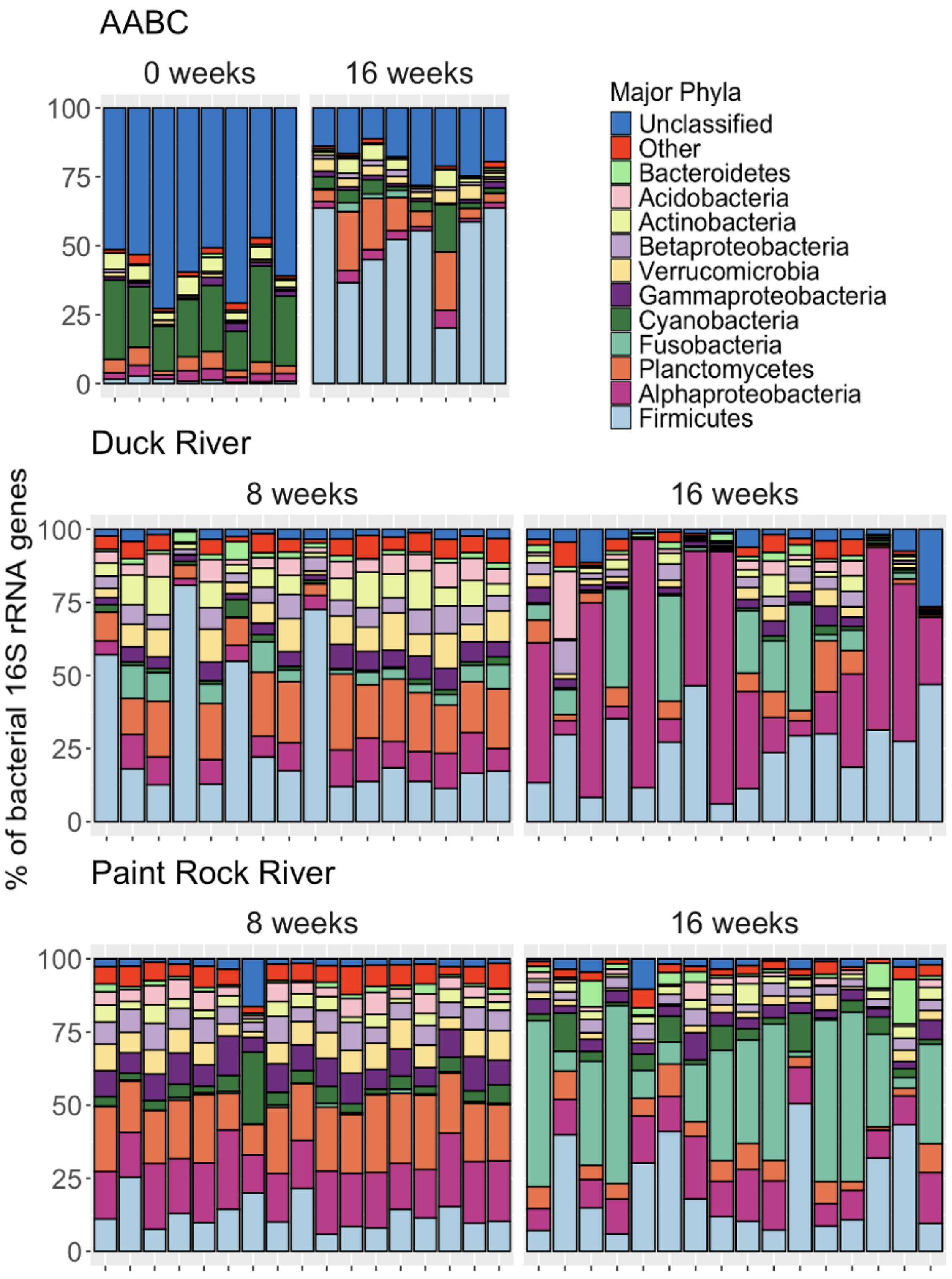

Figure S3. Major bacterial phyla, as determined from percentage of 16S rRNA gene sequences recovered, detected in the gut bacterial communities of hatchery-propagated *Lampsilis ovata* from the Alabama Aquatic Biodiversity Center (AABC) and placed in the Duck and Paint Rock rivers of the Tennessee River Basin. Each bar represents one individual and grouped based on mussel age/time in river (i.e. 0 weeks, 8 weeks, or 16 weeks).

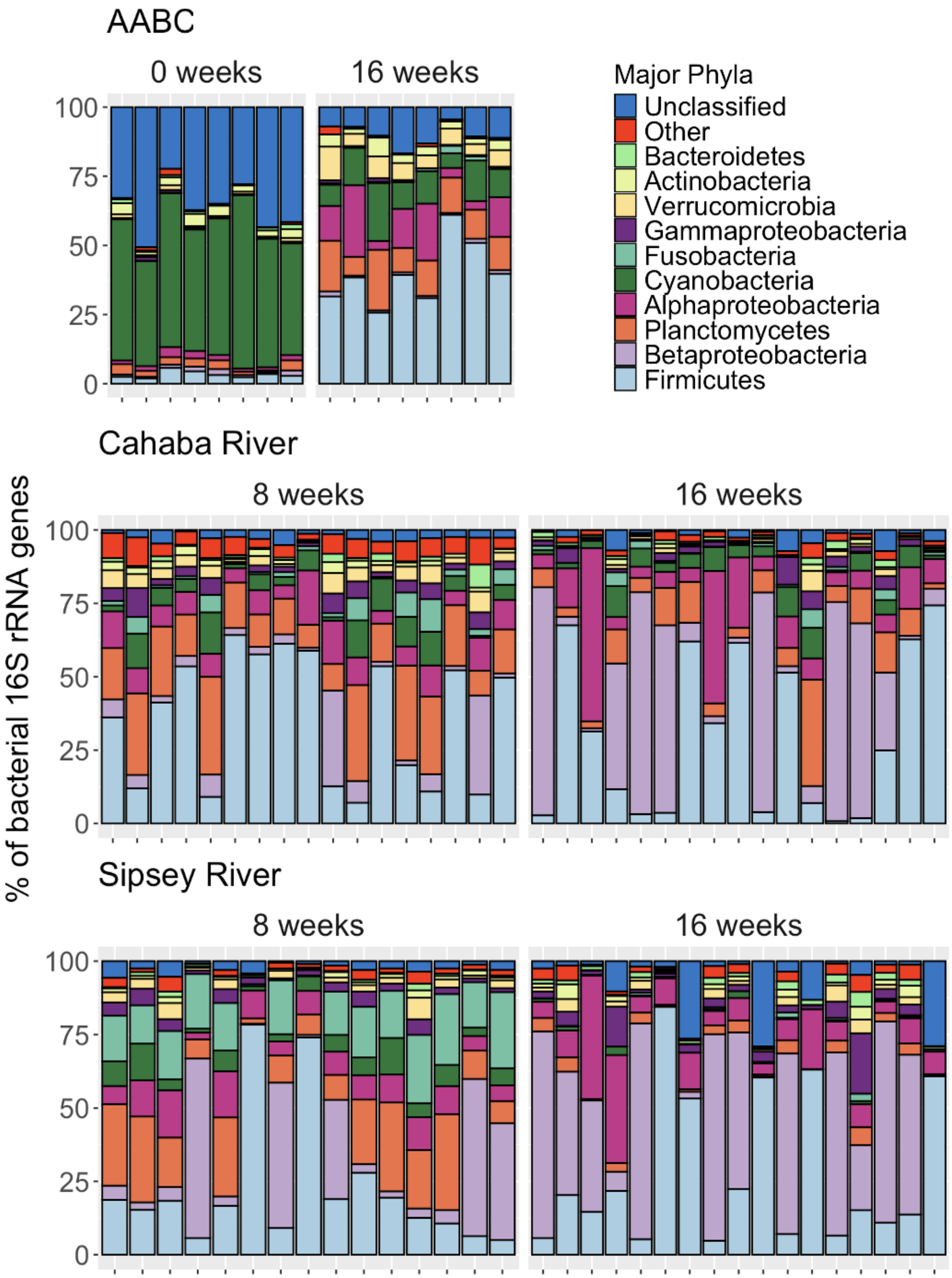

Figure S4. Major bacterial phyla, as determined from percentage of 16S rRNA gene sequences recovered, detected in the gut bacterial communities of hatchery-propagated *Lampsilis ornata* from the Alabama Aquatic Biodiversity Center (AABC) and placed in the Cahaba and Sipsey rivers of the Mobile River Basin. Each bar represents one individual and grouped based on mussel age/time in river (i.e. 0 weeks, 8 weeks, or 16 weeks).

Table S1. The core gut microbiome of 16 week and reciprocally transplanted *Lampsilis ovata* and *L. ornata* placed into the Duck (D) and Paint Rock (PR) rivers of the Tennessee River Basin and the Cahaba (C) and Sipsey (S) rivers of the Mobile River Basin. ASVs listed comprised >1% of all sequences detected in the gut of each mussel and are ordered based on relative abundance within each group. Frequency was determined from the number of individual gut samples within each group that yielded that ASV. Identifications for each ASV were made to the phylum level followed by the corresponding finest classified taxonomy possible in parentheses.

River	Group	ASV	Taxonomic Classification	Relative Frequency	Absolute Frequency
**Duck River (*L. ovata*)**	16-week	ASV 12	Alphaproteobacteria	93.8%	15/16
		ASV 6	Firmicutes (Bacilli)	93.8%	15/18
		ASV 2	Firmicutes (Bacilli)	87.5%	14/16
		ASV 3	Fusobacteria (*Cetobacterium*)	68.8%	11/16
		ASV 317	Firmicutes (*Clostridium chauvoei*)	43.8%	7/16
	PR-D transplanted	ASV 2	Firmicutes (Bacilli)	93.3%	14/15
		ASV 6	Firmicutes (Bacilli)	86.7%	13/15
		ASV 3	Fusobacteria (*Cetobacterium*)	60.0%	9/15
		ASV 703	Firmicutes (Clostridiales)	40.0%	6/15
		ASV 317	Firmicutes (*Clostridium chauvoei*)	40.0%	6/15
**Paint Rock River (*L. ovata*)**	16-week	ASV 42	Alphaproteobacteria (*Methylocystis*)	100%	16/16
		ASV 29	Alphaproteobacteria (Rhizobiales)	100%	16/16
		ASV 3	Fusobacteria (*Cetobacterium*)	100%	16/16
		ASV 55	Planctomycetes (Pirellulales)	81.3%	13/16
		ASV 6	Firmicutes (Bacilli)	75.0%	12/16
	D-PR transplanted	ASV 3	Fusobacteria (*Cetobacterium*)	92.9%	13/14
		ASV 29	Alphaproteobacteria (Rhizobiales)	64.3%	9/14
		ASV 12	Alphaproteobacteria	64.3%	9/14
		ASV 55	Planctomycetes (Pirellulales)	57.1%	8/14
		ASV 42	Alphaproteobacteria (*Methylocystis*)	57.1%	8/14
**Cahaba River (*L. ornata*)**	16-week	ASV 10	Planctomycetes (*Fimbriiglobus*)	94.1%	16/17
		ASV 26	Cyanobacteria	82.4%	14/17
		ASV 12	Alphaproteobacteria	82.4%	14/17
		ASV 1	Proteobacteria (Burkholderiales)	76.5%	13/17
		ASV 55	Planctomycetes (Pirellulales)	52.9%	9/17
	S-C transplanted	ASV 1	Proteobacteria (Burkholderiales)	100%	13/13
		ASV 2	Firmicutes (Bacilli)	84.6%	11/13
		ASV 10	Planctomycetes (*Fimbriiglobus*)	76.9%	10/13
		ASV 26	Cyanobacteria	69.2%	9/13
		ASV 32	Planctomycetes (Gemmataceae)	61.5%	8/13
**Sipsey River (*L. ornata*)**	16-week	ASV 51	Firmicutes (*Romboutsia sedimentorum*)	82.4%	14/17
		ASV 1	Proteobacteria (Burkholderiales)	70.6%	12/17
		ASV 66	Firmicutes	64.7%	11/17
		ASV 12	Alphaproteobacteria	58.8%	10/17
		ASV 317	Firmicutes (*Clostridium chauvoei*)	52.9%	9/17
	C-S transplanted	ASV 1	Proteobacteria (Burkholderiales)	100%	15/15
		ASV 66	Firmicutes	93.3%	14/15
		ASV 239	Bacteroidetes (Flavobacterium)	66.7%	10/15
		ASV 51	Firmicutes (*Romboutsia sedimentorum*)	66.7%	10/15
		ASV 12	Alphaproteobacteria	60.0%	9/15
